# Molecular Targets and Emerging Therapeutic Options for Uterine Leiomyosarcoma

**DOI:** 10.1155/2016/7018106

**Published:** 2016-09-19

**Authors:** Heather Miller, Chiemeka Ike, Jennifer Parma, Ramya P. Masand, Claire M. Mach, Matthew L. Anderson

**Affiliations:** ^1^Department of Obstetrics & Gynecology, Baylor College of Medicine, Houston, TX 77030, USA; ^2^College of Pharmacy, University of Houston, Houston, TX 77030, USA; ^3^Department of Pathology & Immunology, Baylor College of Medicine, Houston, TX 77030, USA; ^4^Dan L Duncan Cancer Center, Baylor College of Medicine, Houston, TX 77030, USA

## Abstract

Uterine leiomyosarcoma (uLMS) is an aggressive malignancy characterized by its early metastasis, high rates of recurrence, and poor prognosis. Multiple obstacles complicate the clinical management of uLMS. These include the fact that most uLMS are typically identified only after a woman has undergone hysterectomy or myomectomy, the limited efficacy of adjuvant therapy for early stage disease, and the poor response of metastatic disease to current treatments. Here, we discuss recent insights into the molecular basis of uLMS and discuss emerging options for its clinical management. Particular attention is given to the biologic basis of these strategies with the goal of understanding the rationale motivating their use.

## 1. Introduction

Uterine leiomyosarcoma (uLMS) is an aggressive cancer characterized by its poor prognosis and high rates of recurrence [1, 2]. Although fewer than 3,000 women are diagnosed with this disease in the United States each year, uLMS is the most common sarcoma arising in the genital tract of reproductive-aged women [3, 4].

A number of long-standing challenges complicate clinical management of uLMS. Perhaps one of the most important of these challenges is the fact that uLMS are typically diagnosed only after a woman has undergone hysterectomy or myomectomy. This problem occurs for a number of reasons. The most important of these reasons is that strategies used to identify other types of uterine cancer, such as endometrial biopsy, are not useful for diagnosing this disease [5, 6]. Currently, uLMS are diagnosed on the basis of the histologic identification of a high mitotic count (>10 mitotic figures/10 high power fields) and the presence of coagulative tumor necrosis and moderate to severe cytologic atypia [7]. Unfortunately, these features cannot be sufficiently evaluated by small volume core biopsies or needle aspirations to differentiate malignant from nonmalignant myometrial tissue. In addition, symptoms associated with uLMS, such as irregular vaginal bleeding or pelvic pain, are nonspecific and frequently caused by multiple, more common but benign etiologies [8]. A rapidly growing myometrial mass is often presumed to be pathognomonic, although existing data fail to support this belief [9].

Recognizing a clear area of clinical need, investigators have explored the utility of different imaging modalities to distinguish uLMS from benign leiomyomas preoperatively. Although a number of specific radiographic features, such as infiltrative margins, have been associated with uLMS visualized by magnetic resonance imaging (MRI), these features are not found frequently enough to allow routine use of MRI to prospectively distinguish benign from malignant myometrial masses [10]. Furthermore, there are no radiographic features capable of reliably distinguishing uLMS from benign myometrial masses by pelvic ultrasonography, computed tomography (CT), or positron emission tomography (PET).

A second key clinical challenge complicating management of uLMS is the need for effective adjuvant therapy following hysterectomy. Most uLMS (68%) are diagnosed as a solitary mass grossly confined to the uterus, which is defined as stage I disease according to the 2009 revised FIGO criteria [11]. Recurrence rates even for early stage disease are high, ranging from 53 to 71% [12, 13]. As a result, three-year survival for FIGO stage I uLMS is estimated to be only 52% [1]. Unfortunately, surgical staging is largely unable to identify women at risk of experiencing a recurrence of their disease. In the absence of grossly visible metastases, routine pelvic and para-aortic lymphadenectomy identifies microscopic metastases in only 2-3% of cases [14, 15]. Routine oophorectomy similarly fails to provide prognostic insight. In fact, oophorectomy has been associated with worse overall survival, although data addressing this issue remains conflicted [16].

Given the high recurrence rates associated with early stage disease, adjuvant therapy is frequently administered to women who have recently undergone hysterectomy for uLMS. Multiple, early retrospective studies promoted the use of adjuvant radiotherapy to reduce the incidence of disease recurrence [17, 18]. However, at least one recent prospective randomized control trial has failed to demonstrate any improvement in progression-free or overall survival for women with early stage uterine sarcomas including uLMS treated with radiotherapy [1, 19]. As a result, use of adjuvant radiotherapy has largely been abandoned. Several large retrospective studies have suggested that adjuvant chemotherapy may also provide little benefit. The role of adriamycin as adjuvant therapy following surgical management of stage I or II disease has been studied with no difference in PFS or OS observed [20]. More recently, a phase III clinical trial compared the uses of doxorubicin, ifosfamide, and cisplatin as adjuvant therapy with and without radiotherapy for uterine sarcoma. Data from this study revealed a slight increase in 3-year disease-free survival in subjects who received both chemotherapy and radiotherapy [21]. Another phase II clinical trial examined the efficacy of adjuvant gemcitabine and docetaxel in patients with completely resected stage I and II disease. The outcome of this study demonstrated 57% progression-free survival (PFS) at 3 years, which was significantly greater than the 35% PFS observed at 3 years among historical controls [11, 22]. Despite the fact that each of these studies demonstrated modest improvements in PFS associated with the use of adjuvant chemotherapy, none of the regimens studied to date have yet to emerge as a consistent standard.

A final challenge is the treatment of advanced stage and/or recurrent uLMS. Similar to early stage disease, doxorubicin with or without ifosfamide has been historically used as frontline therapy for advanced or recurrent disease. However, response rates to doxorubicin-based strategies range from 15 to 30% [23]. A more recent phase II study has shown that the combination of gemcitabine and docetaxel may be more effective, with response rates reported to be as high as 36% [24, 25]. The use of gemcitabine and docetaxel as first line therapy has been prospectively compared to doxorubicin as part of a phase III randomized trial (GeDDIS) enrolling patients with metastatic soft tissue sarcomas. Although similar progression-free survival rates were observed in both treatment arms, subjects treated with doxorubicin experienced significantly less toxicity. Study investigators concluded that doxorubicin should remain first line treatment for patients with metastatic soft tissue sarcomas [26].

**Figure 1:**
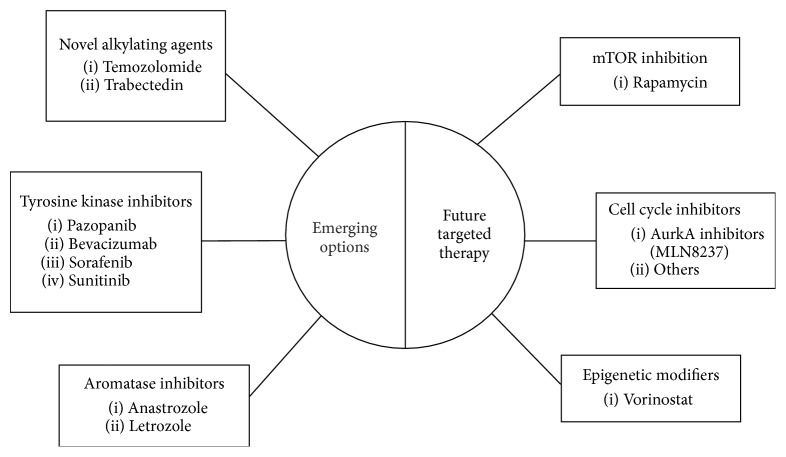
Graphic summary of emerging and potential future targeted therapy for uLMS.

Given these challenges, there is an overwhelming need to develop more effective strategies for the diagnosis and treatment of uLMS. Solutions will be made more difficult by the fact that uLMS now appears to be a unique biologic entity which responds differently to treatments than leiomyosarcomas arising at extrauterine sites [27–30]. Here, we review recent advances in the clinical management of uterine leiomyosarcoma, emphasizing the biologic basis of emerging therapeutic options (Figure 1).

## 2. Novel Targeted Therapies

### 2.1. Tyrosine Kinase Inhibitors

Uterine leiomyosarcoma is a highly vascular cancer that expresses significantly greater levels of vascular endothelial growth factor (VEGF) expression than many other tumors [31]. The overexpression of VEGF in uLMS has been shown to correlate with higher tumor grade, disease metastasis, and decreased survival [32, 33]. Although multiple antiangiogenic agents are now available for clinical use, the response of uLMS to many of these agents, such as bevacizumab, has been disappointing [34, 35]. A recent phase III clinical trial compared treatment with gemcitabine plus docetaxel with or without bevacizumab as first line therapy for advanced stage or recurrent uLMS. The results of this study failed to demonstrate any improvement in either progression-free or overall survival for patients who received bevacizumab [35].

More recently, results of randomized clinical trials have shown that a novel multityrosine kinase inhibitor known as pazopanib may be effective in treating soft tissue sarcomas. Pazopanib differs from bevacizumab in that it targets and inhibits not only the inherent tyrosine kinase activity of the VEGF receptor, but also those of the PDGF receptor and stem cell factor receptor (c-kit) [36]. Overexpression of EGFR, PDGFR-*α*, PDGFR-*β*, and PDGF-B has been reported to be a feature of uLMS [29]. Furthermore, levels of PDGF-B expression in uLMS specimens from the same study have been shown to correlate with outcome [29]. These observations suggest that the activation of PDGF-regulated pathways plays a particularly important role in promoting the growth and metastasis of uLMS.

Recently, the European Organization for the Research and Treatment of Cancer study concluded a phase III clinical trial entitled PALETTE, which compared pazopanib to placebo as a treatment for soft tissue sarcomas. Patients diagnosed with both uterine and nonuterine leiomyosarcomas were enrolled. In this multicenter, double blind study, patients with progressive disease were randomized to receive either pazopanib 800 mg once daily or placebo. Investigators reported a 3-month increase in progression-free survival for patients who received pazopanib [37].

Regorafenib is a multikinase inhibitor with documented efficacy for the treatment of gastrointestinal stromal tumors. Regorafenib has also recently been studied as a treatment for LMS [38]. A phase II double-blinded, randomized control trial compared regorafenib to placebo in patients with metastatic soft tissue sarcomas previously treated with anthracycline chemotherapy. In the subgroup of LMS patients enrolled in this study, PFS was found to be 4.0 months in patients receiving regorafenib compared to 1.9 months in the placebo arm. The most common adverse events noted were hypertension, skin toxicity, asthenia, and diarrhea [39, 40].

Olaratumab is a human antiplatelet derived growth factor receptor alpha monoclonal antibody that has been shown to have antitumor effects in human sarcoma xenografts [41]. Olaratumab specifically binds PDGFR*α* and blocks activation of this receptor by PDGF-AA, PDGF-BB, and PDGF-CC. A phase II randomized control trial compared the use of doxorubicin with and without olaratumab in patients with soft tissue sarcoma who had not previously been treated with an anthracycline. Subjects treated with doxorubicin and olaratumab had PFS of 6.6 months compared to 4.1 months with doxorubicin alone. Overall survival was better for subjects treated with the combination of doxorubicin and olaratumab (26.5 months compared to 14.7 months in those treated with doxorubicin alone). Neutropenia, mucositis, nausea, vomiting, and diarrhea each occurred more commonly in those subjects treated with olaratumab [42]. A phase III clinical trial is currently ongoing.

### 2.2. Antihormonal Agents

Reflecting their origin in the female reproductive tract, gynecologic sarcomas often express receptors for estrogen and progesterone. When examined immunohistochemically, 25–60% of uLMS have been found to express estrogen receptor (ER), while 35–60% of cases express progesterone receptor (PR) [43, 44].

Multiple studies have shown that estrogen and progesterone receptor expression in uLMS correlates with improved prognosis [43, 45, 46]. However, at least one group has suggested that activation of PR may serve to promote the growth of uterine smooth muscle [47]. Transgenic expression of Simian Virus 40 large T antigen driven by the promoter for oviduct-specific glycoprotein has been recently shown to result in the development of leiomyosarcoma [48, 49]. Of note, these transgenic animals develop LMS only under the influence of estrogen. These findings suggest that circulating levels of ligand-specific ER activation play an important role in tumor development.

Options currently available to inhibit activity at both the estrogen (ER) and progesterone (PR) receptors include both direct receptor antagonists as well as aromatase inhibitors. A number of studies have also examined the efficacy of direct ER antagonists such as tamoxifen. However, response to tamoxifen appears quite limited [44]. Aromatase inhibitors are orally available medications that decrease circulating levels of estrogen by targeting the peripheral conversion of testosterone and androstenedione to estrogen in adipose tissue. Retrospective studies have shown objective response rates and improvement in progression-free survival in patients with uLMS treated with aromatase inhibitors such as anastrozole or letrozole [50, 51]. A phase II clinical trial was conducted that examined the effect of letrozole 2.5 mg daily in 27 patients with unresectable uLMS, most of whom had already received cytotoxic treatment. All patients had confirmed ER/PR expression as evidenced by immunohistochemical staining. Stable disease was observed in 54% of patients receiving letrozole, with no evidence of disease progression at 12 weeks for 46% of patients [52].

Given the central role of both ER and PR in regulating the growth and remodeling of uterine smooth muscle, future work aimed at understanding the potential role of targeting steroid hormone receptors and/or their coactivators or corepressors as therapeutic options in uLMS are clearly warranted. Better understanding of the role of ER and PR in this disease may also help to decipher the circumstances under which oophorectomy may benefit the outcome.

### 2.3. Next Generation Alkylating Agents

As mentioned above, doxorubicin and other alkylating agents have been one of the primary approaches used to treat both metastatic and recurrent uLMS [23, 53]. Over the past 5 years, use of these agents has declined, with clinicians more frequently favoring the use of other regimens. However, recent success with orally available alkylating agents, such as temozolomide, has precipitated renewed interest in their use in the treatment of soft tissue sarcomas [54, 55]. A small retrospective study of 12 women with unresectable disease compared the efficacy of two dosing regimens for temozolomide in uLMS. Response rates appeared higher in patients treated with a bolus regimen (14%) rather than with a continuous dosing regimen (8%) [56]. A recent report has found that O6-methylguanine-DNA methyltransferase (MGMT) gene promoter hypermethylation is associated with response of glioblastoma to temozolomide therapy and may be a key factor determining a response to this agent. In a number of cancers, promoter hypermethylation has been shown to result in decreased expression of MGMT and gene as well as diminished DNA repair activity, rendering cells more susceptible to alkylating agents [57]. Hypermethylation of the MGMT gene promoter has also been observed in uterine sarcomas and thus could serve as a potential marker to identify patients who would benefit from temozolomide use [58, 59].

Trabectedin is another novel alkylating agent that has been recently approved in the United States for use in treating unresectable uLMS that have progressed despite receiving a standard cytotoxic chemotherapy regimen, which included an anthracycline. Trabectedin is a marine-derived alkaloid that interacts with the minor groove of DNA, leading to apoptosis [60]. Studies have examined the use of trabectedin alone and in combination with other chemotherapeutic agents. A phase II study examining the use of trabectedin as a single agent in chemotherapy naïve patients with advanced uLMS reported a 10% partial response rate [61]. However, trabectedin must be continued in absence of progression of disease, as discontinuation of the drug is associated with decreased progression-free survival. Another phase II study examined the efficacy of first line therapy with the combination of trabectedin and doxorubicin and reported a partial response rate of 60% [62]. The role of MGMT promoter hypermethylation in determining clinical responses to trabectedin is not currently known.

### 2.4. mTOR Inhibition

mTOR is a serine/threonine kinase involved in cell growth, proliferation, survival, and metabolism [63]. mTOR activation has been shown to contribute to uLMS cell growth and cell cycle progression. Specifically, loss of PTEN (phosphatase and tensin homolog) results in AKT-mTOR activation. In transgenic mice, inactivation or loss of PTEN leads to the development of uLMS [64, 65]. Rapamycin blocks the mTOR pathway and has been shown to have antitumor effects in preclinical models and has been shown to inhibit growth and cell cycle progression in uLMS cell lines [66, 67]. In PTEN knockout mice, everolimus, a rapamycin derivative, was shown to decelerate tumor growth [64]. mTOR inhibitors have been used clinically for treatment of soft tissue sarcomas [68]. Additional work is needed to determine the efficacy of mTOR inhibitors in uLMS specifically.

## 3. Novel Targets with Potential for Future Utility

### 3.1. Cell Cycle Inhibitors

In contrast to tumors, such as gastrointestinal stromal sarcomas (GIST), leiomyosarcoma regardless of its anatomic origin is considered genetically heterogeneous. This means that dominant driver mutations for uLMS have not been identified. A number of recent studies have examined patterns of gene expression in LMS of both uterine and nonuterine origin. It is clear from this work that the robust overexpression of gene products regulating the G2-M phase of the cell cycle is single predominant feature of these tumors [69, 70]. In one recent study, 26 of the 50 gene products most overexpressed when specimens of uLMS were compared to either healthy myometrium or benign uterine leiomyomas which played a role in regulation of the G2-M checkpoint [70]. Aurora kinase-A (AurkA) is a serine-threonine kinase involved in centrosome function and spindle assembly and has been shown to be highly overexpressed in uLMS. Use of MLN82237, an oral Aurk-A inhibitor, has been found to inhibit growth and metastasis of uLMS both* in vitro* and* in vivo*, using xenograft models [69]. Additional therapeutic benefit was observed when MLN82237 was combined with mTOR inhibition, consistent with AurkA's ability to cross-regulate mTOR pathway activity [67, 71]. A small, phase II clinical trial has examined the efficacy of MLN8237 (alisertib) monotherapy in 21 women with recurrent or persistent uLMS. All subjects in this study had received 1 to 2 prior cytotoxic regimens. Although no objective responses were reported, stable disease was observed in 38% of patients with mean progression-free and overall survival of 1.7 and 14.5 months, respectively [72]. Although the investigators for this trial concluded that alisertib did not demonstrate meaningful clinical activity against uLMS, it is possible that better efficacy will be observed when used in combination with mTOR inhibitors or even other types of cytotoxic or targeted agents.

### 3.2. Epigenetic Modifiers

Histone deacetylases (HDAC) are enzymes, which regulate patterns of gene expression modulating cell growth and apoptosis by removing acetyl moieties from histones [73, 74]. HDAC expression has been shown to be consistently elevated in endometrial stromal sarcomas [75]. Data has also shown that HDAC inhibitors can enhance chemotherapy-induced apoptosis and reduce sarcoma tumor volume in preclinical models [76]. Vorinostat is an oral HDAC inhibitor that has already been FDA approved for the treatment of cutaneous T cell lymphoma [77]. Studies have shown that vorinostat suppresses tumor growth in uterine sarcoma cells [76]. Given this preclinical data, HDAC inhibitors are being studied for targeted therapy in uterine sarcomas. A phase I trial evaluated the use of a HDAC inhibitor, abexinostat, in combination with doxorubicin for patients with metastatic sarcoma and showed manageable toxicities [78]. Further studies are needed to evaluate the efficacy of HDAC inhibitors in combination with cytotoxic agents and in uLMS specifically.

## 4. Conclusion

Management options for uLMS have evolved rapidly over the past several years with the advent of specific targeted therapies. These options have given patients new hope for improving their outcome. Given that uLMS likely represents a unique subset, distinct from extrauterine leiomyosarcomas, future therapeutic advances for this aggressive disease will need to consider its unique biologic basis.
